# In the Hospitals Sixty Years Ago

**Published:** 1897-06-05

**Authors:** 


### Modern Sociology.

IN THE HOSPITALS SIXTY YEARS AGO.

Entering as we are now into the month of June, a month devoted to loyal celebrations, it is difficult to avoid looking backward to times gone by, and asking, in regard to things we see around us now, what was their condition when Her Majesty ascended the throne sixty years ago?

The changes in medicine have been so great, even within the active life of middle-aged men, that we may well believe how greatly the hospitals have changed since the days when they were "walked" by men who are now old; and few things can be more interesting to those who are familiar with the inner working of the hospitals of to-day than to listen to the tales told of what they were like at the beginning of the reign.

When we consider that we are looking back to the era of flint and steel and snuffers, we need not be surprised to find how different were the wards of our hospitals from what they are at the present time. The wards, we are told, were kept tolerably clean by dint of constant scrubbing—scrubbing so constant, in fact, that one never entered a ward without finding a part of it wet from the recent washing.

This cleaning was done by the nurses, who assisted in doing all the menial work which the ward required. Then a certain amount of cooking was done in the wards, the making of broth and barley water being especially common; and as poultices constituted the ordinary dressing a continuous manufacture of linseed poultices used to be in progress. Dr. Wilks says that even now his earliest recollections of Guy's Hospital have ever since brought before his mental smell a faint compound odour of boiled mutton and sour poultices.

One of the most marked changes in the hospitals between then and now is the much greater civility and consideration with which the patients are now treated. There seems to have been a considerable difference in this respect in early days between the physicians and the surgeons. Dr. Wilks* tells us that the latter with his class went stamping about the wards with their hats on, while the former proceeded much more quietly. Strong language, also, was not uncommon, and a surgeon while operating on his patient has in those days been heard to exclaim, "D——n you, lie still," a thing that can scarcely be credited at the present day. We cannot here enter into the vast changes which have taken place during the past half-century in the regions of medicine and pathology, or speak of the new diseases which have been discovered, the new instruments which have been contrived, the new modes of investigation which have come into use. For such matters we may refer to the *Practitioner* for June, where an admirable review will be found of the progress of medicine during the past sixty years. But we must recall the fact that at the beginning of the reign Bright's disease was a new thing, all the three forms of continued fever were lumped together, bacteriology was utterly unknown, and antisepsis was unthought of, there were no anæsthetics, no hypodermic syringes, no aspirators, no ophthalmoscopes, no laryngoscopes, no cystoscopes, no sphygmographs, and even no clinical thermometers.

It is in surgery, however, that the greatest differences can be found between then and now. When we consider the importance now attached to absolute cleanliness in all that concerns operative surgery, nothing can seem more horrible than the description given* of the rooms in which operations were performed at that time: "Sixty years ago the operating theatre was the dirtiest room in the hospital. There was no apparent reason why it should be otherwise. It was as clean as a butcher's shambles, and that was sufficient to satisfy the fastidious. The surgeon operated in the dirtiest coat in his possession—a coat stiff with blood and animal filth. He was as proud of this blood-stained rag as a peer of ancient lineage may be of his faded ceremonial robes." Hæmorrhage was not easily controlled in those days, and blood was plentiful. "The expectant patient received scant attention as he lay upon the table, with his eyes turned to the rafters, listening acutely for that footstep which would tell him that the hideous moment had come. He could relieve the tedium of waiting by watching the sawdust being thrown down to catch his blood, and by listening to the graceful converse of the medical student of the day. Finally, into the hushed arena would step the great surgeon, and about his entrance would centre some such suppressed thrill as would attend a great matador when he stepped into the ring. There was something heroic about the surgeon of sixty years ago. . . . Every movement of his terrible blade through the quivering flesh was made horrible by moans and shrieks, by entreaties and curses." How different all this is now!

Needless to say, everything in those days gave way to speed. The only way by which a humane surgeon could lessen the sufferings of his patient was to study celerity, and, whatever else might happen, to be quick about his work. The slow and careful operations of to-day were entirely unknown. Who now ever sees the watch pulled out to time the operator, yet the watch was the measure of the operator's reputation in the times gone by.

It is probable that the horrors of the operating-room had something to do with the low status of the nurse in those days. There was a feeling, says Miss Wood, almost amounting to repulsion at the thought of entering a hospital as a nurse; it was considered indecent, unwomanly, revolting—and indeed there was much in our hospitals at that period to justify these feelings. It was only by degrees that the decencies and refinements of nursing, now the invariable custom, but then introduced in the teeth of opposition on the part of the authorities, were accepted, and this advance has been due to the nursing sisterhoods, bodies of religious women who, in King's College, Charing Cross, and other hospitals, were quietly leavening the practice of nursing.

We think, however, that other influences were at work, both in regard to nursing and to the *personnel* of the surgical staff. There can be no doubt that the introduction of anæsthesia not only opened up the profession of surgery to men of science and sensibility, who would have shrunk from the horrors of the operating-room of old, but it also made it possible for women of more tender mould to undertake the work of nurses.

* *Practitioner*, June, 1897.

* *Practitioner*, June, 1897.

